# Usefulness and safety of new ultrasmall-diameter colonoscope for cases with difficult insertion: a retrospective study

**DOI:** 10.1038/s41598-024-72689-1

**Published:** 2024-09-14

**Authors:** Rie Terada, Ryoji Ichijima, Aya Iwao, Hiroshi Kinebuchi, Yuta Okada, Tomomi Sugita, Kanako Ogura, Akiko Haruta, Hirofumi Kogure

**Affiliations:** 1 Division of Gastroenterology and Hepatology, Department of Medicine, Nihon University School of Medicine, 30-1, Oyaguchi Kami-cho, Itabashi-ku, Tokyo, 173-8610 Japan; 2 Health Management Center, Toranomon Hospital, Tokyo, Japan

**Keywords:** Adenoma detection rate, Colorectal cancer, Linked color imaging, Blue laser imaging, Ultrathin endoscopes, Colonoscopy, Gastrointestinal system

## Abstract

Colonoscopies are widely available, but there are cases where insertion can be difficult, even for experienced endoscopists. EC-760XP/L, a new ultrasmall-diameter long scope, may be useful in such cases. This single-center retrospective study included 39 cases where colonoscope insertion was difficult even when previously conducted by an experienced endoscopist. The primary outcome was the cecal intubation time using EC-760XP/L compared to the time used in a previous examination with a standard scope. The secondary outcomes were the cecum intubation rate, intestinal cleanliness level, adenoma detection rate, polyp detection rate, sedative use rate, occurrence of adverse events, and pain experience. A comparison of cecal intubation times between EC-760XP/L and the standard scope showed that insertion times were significantly lower with EC-760XP/L (9.5 min) compared to the standard scope (19 min) (*p* < 0.01). The standard scope achieved cecal intubation in 30 cases (76.9%), whereas EC-760XP/L reached the cecum in all cases (*p* < 0.01). Pain was observed in 3 cases (8.3%) with the EC-760XP/L, which was significantly lower than the 22 cases (56.4%) with the standard scope (*p* < 0.01). In conclusion, EC-760XP/L proved to be useful in cases where colonoscope insertion was difficult.

## Introduction

Colorectal cancer is ranked third in terms of morbidity and second in terms of mortality rate worldwide^1^. It has been suggested that the removal of adenomatous colonic polyps using a colonoscope is an effective means of preventing colorectal cancer^2–4^. Therefore, colonoscopies are an essential examination method for preventing and treating colorectal cancer. However, cases with pronounced inflammation, a history of abdominal surgery, and poor pretreatment may result in longer cecal intubation times, inability to intubate the cecum, or increased pain^5^. Patients who experience extended cecal intubation times or significant pain may be less inclined to consent to future examinations. Additionally, in cases where cecal intubation is unsuccessful, there is a concern that colorectal cancer may develop in the regions not examined. Quality indicators of colonoscopy include the adenoma detection rate (ADR) and cecal intubation rate, and cases where intubation is difficult may decrease these indicators^6–10^. Even skilled endoscopists occasionally encounter difficult insertions. While sedation during endoscopy can alleviate pain, it must be administered with caution in elderly patients and those with underlying conditions due to potential adverse events, including respiratory depression and hypotension^11,12^. Various insertion methods and pretreatment methods have been previously reported, but some patients still experience pain during insertion. The use of ultrathin endoscopes has been reported to improve the ileocecal region intubation rate and reduce patient pain^5^.

**Fig. 1:**
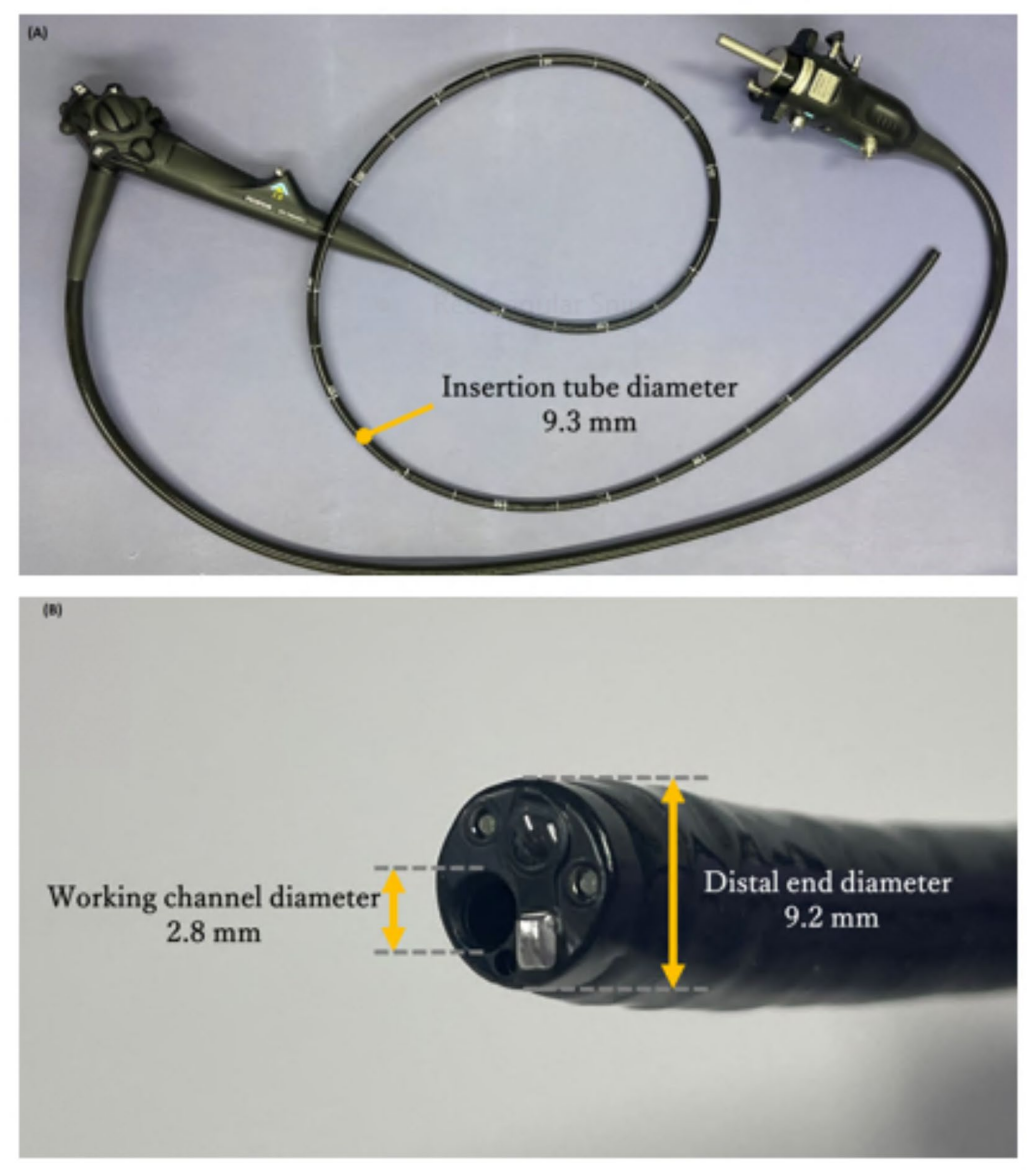
The image of EC-760XP/L. (**a**) The whole image of EC-760XP/L. (**b**) The zoomed image of the distal part of the EC-760XP/L with lens, working channel.

EC-760XP/L (FUJIFILM Corporation, Tokyo, Japan), a new ultra-small-diameter long scope with a distal end diameter 9.2 mm, an insertion tube diameter of 9.3 mm, with a working channel diameter of 2.8 mm and an effective length of 1690 mm, appears highly effective in cases with insertion difficulties (Fig. 1a, b).

Unlike conventional small-diameter endoscopes with limited sub-water supply functions, EC-760XP/L is equipped with a sub-water supply function, enhancing its utility in inadequate pretreatment cases and managing post-procedural bleeding. It also supports image-enhanced observation techniques such as Linked Color Imaging (LCI) and Blue Laser Imaging (BLI), along with the CAD EYE system incorporating artificial intelligence technology, raising expectations for improved ADR and diagnostic accuracy^13–16^. As there are no prior reports on EC-760XP/L, this study aims to evaluate its usefulness and safety in cases with insertion difficulties encountered by endoscopists.

## Methods

### Study design

This was a single-center, retrospective study. The study was registered with the University Hospital Medical Information Network (UMIN 000052830) and conducted in accordance with the Helsinki Convention.

Written informed consent was obtained from all patients before colonoscopy. This study received approval from the review board of Nihon University School of Medicine.

### Inclusion and exclusion criteria

**Fig. 2:**
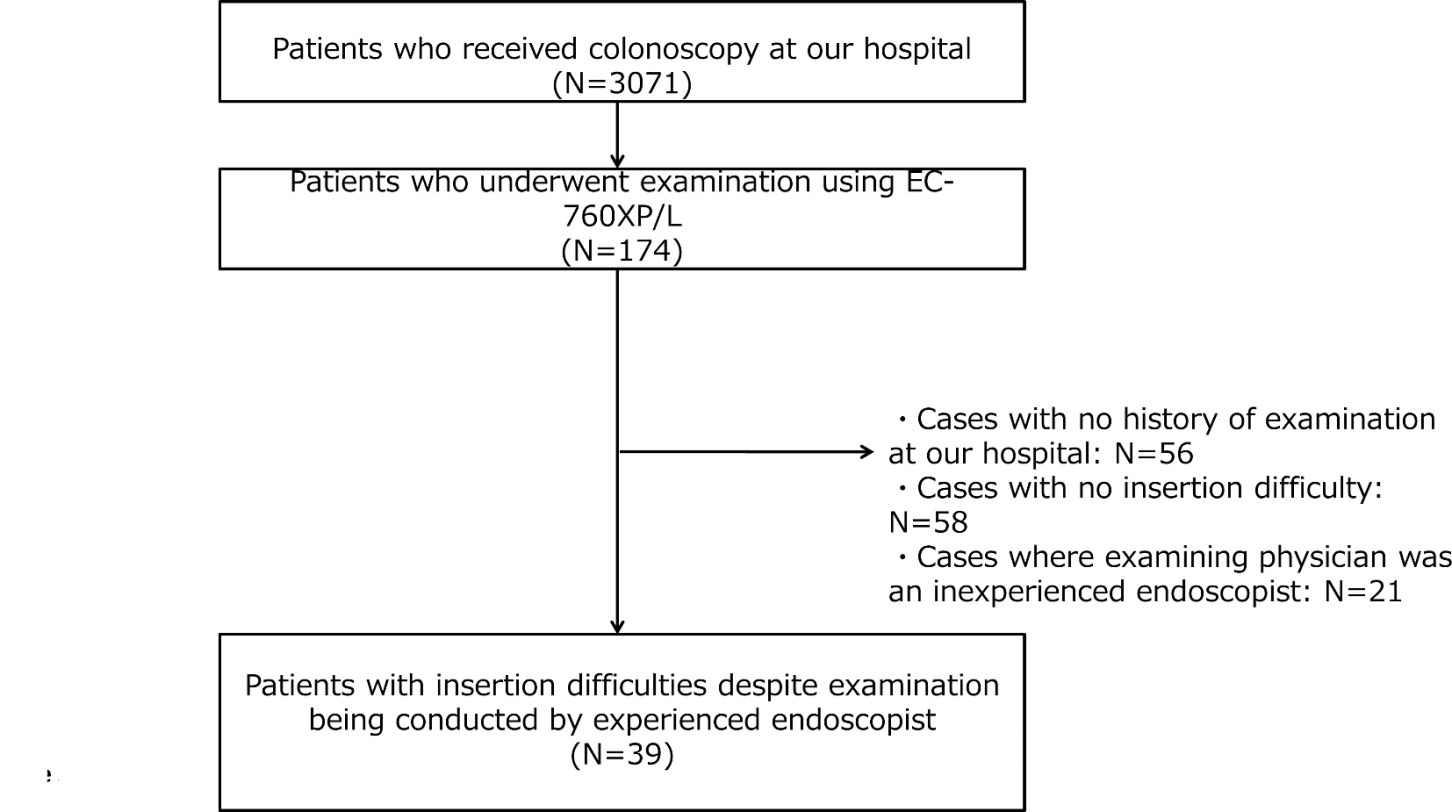
Flowchart of the patient selection process.

Figure 2 presents the flow chart of this study. We included cases where colonoscope insertion was difficult despite a previous examination by an experienced endoscopist. Of the 3,071 patients who underwent colonoscopies between June 2022 and October 2023, 174 were examined using EC-760XP/L scope. From this group, we excluded the following: 56 cases without a history of colonoscopy at our hospital, 58 cases where the previous endoscope insertion was not difficult, and 21 cases where the previous or current endoscopist was considered inexperienced. Consequently, a total of 39 subjects were included in the analysis.

### Endoscopic procedure

All patients undergoing colonoscopies had not eaten since 21:00 the day prior. Patients ingested sodium picosulfate (provided by Tsuruhara Pharmaceutical Co. Ltd., Osaka, Japan) orally before bed, the night before the examination. On the day of the examination, they used MoviPrep (EA Pharma Co., Tokyo, Japan) or Niflec (EA Pharma Co.) as a laxative. EC-760XP/L was used in the examination for all cases, with CO_2_ insufflation. The operator used butylscopolamine or glucagon as an antispasmodic, depending on the patient’s underlying disease and preference. Midazolam was used as a sedative. Before initiating the examination, the physician confirmed the patient’s preferences. Medications were administered before the examination or upon the patient expressing pain.

### Definition

Endoscope insertion difficulties were defined as cases where cecal intubation could not be achieved in the endoscopy conducted at our hospital or where the cecal intubation time was 15 min or longer despite being examined by an experienced endoscopist using a standard endoscope during the previous endoscopy.

An experienced endoscopist was defined as one with experience in colonoscopy and who was certified by the Japan Gastroenterological Endoscopy Society. Evaluations regarding the presence of pain were determined based on the descriptions of the endoscopist who conducted the examination. The removal time was defined as the time from cecal intubation until the scope was removed and the examination was completed. SD endoscope was defined as one of the following: CF-H290I, CF-HQ290I, PCF-H290I, PCF-H290ZI, PCF-Q260AI, PCF-Q260AZI, PCF-Q260JI, PCF-Q240ZI (Olympus, Tokyo, Japan), and EC-760ZP-V/M (FUJIFILM Corporation, Tokyo, Japan). Evaluations of intestinal cleanliness levels involved the use of the BBPS^17^. A score of ≥ 6 out of 9 was considered good in terms of pretreatment. Adverse events included post examination abdominal pain, nausea, vomiting, and intestinal perforation. We categorized the pain experienced during colonoscopy into three groups: A, little to no pain or mild pain; B, presence of pain but examination could be completed; and C, incomplete colonoscopy due to pain.

### Outcomes

The primary outcome was the cecal intubation time using EC-760XP/L compared to a previous examination with a standard scope. The secondary outcomes were the cecum intubation rate, intestinal cleanliness level, ADR, polyp detection rate (PDR), sedative use rate, occurrence of adverse events, and pain experience.

### Statistical analysis

Descriptive data are expressed as median and interquartile range. Comparisons between groups were conducted using the Wilcoxon signed rank test. The χ-squared test or Fisher’s exact test was used for categorical variables. A p-value of < 0.05 was considered statistically significant. JMP Pro was used for all statistical analyses (version 13.0; JMP Statistical Discovery LLC, Cary, NC, USA).

## Result

### Baseline characteristics

Table 1 shows the participant’s characteristics. The median participant age was 64.0 years (46.5–75.0 years), 15 were male, median height was 161.1 cm (154.0–165.0 cm), median weight was 55.6 kg (50.0–64.0 kg), and median BMI was 21.4 kg/m^2^ (19.4–24.6 kg/m^2^). Insertion of the colonoscope was difficult during previous examination of the 39 participants for the following reasons: cecal intubation difficult, 9 (23.0%); cecal intubation time of ≥ 15 min despite previous examination by an experienced endoscopist using a standard endoscope, 30 (77.0%).

**Table 1:** Participants’ baseline characteristics.

	*N* = 39
Age (IQR), years	67 (57.5–74.3)
Sex, (male / female)	15 (38.5%)/ 24 (61.5%)
Height, cm (IQR)	161.1 (154.0–165.0)
Weight, kg (IQR)	55.6 (50.0–64.0)
BMI, kg/m^2^	21.4 (19.4–24.6)
ASA, n (%) I / II / III	0 (0%) / 37 (94.9%) / 2 (5.1%)
Past History that may make insertion difficult, n (%) Abdominal surgery Diverticulitis	9 (23.0%) 11 (28.2%)
The reasons for difficulty with insertion during the previous examination, n (%) Cecal intubation could not be achieved Cecal intubation time was 15 min or longer	9 (23.0%) 30 (77.0%)

IQR, interquartile range; BMI, body mass index; ASA, American Society of Anesthesiologists physical status grade.

### Endoscopic results

Table 2 shows the therapeutic results of endoscopy. A comparison between EC-760XP/L and a previous standard scope revealed a significant reduction in intubation times: 9.5 min (5.3–12.0 min) for EC-760XP/L versus 19 min (15.9–25.0 min) for the standard scope (*p* < 0.01). While the conventional scope achieved a cecal intubation in 30 cases (76.9%), EC-760XP/L successfully reached the cecum in all cases (*p* < 0.01). No significant difference was observed in ADR and PDR. The median Boston Bowel Preparation Scale (BBPS) values were 9 (6.8–9) for EC-760XP/L and 9 (8–9) for the standard scope, with no significant difference observed. There was no significant difference in sedative use between the two groups. No adverse events associated with the examination were also observed in either group. Regarding pain during the previous colonoscopy using a conventional scope, it was classified as A, B, and C in 16 (41.0%), 19 (48.7%), and 4 (10.3%) participants, respectively. On the other hand, during the colonoscopy using EC-760 XP/L, 36 (92.3%), 3 (7.7%), and 0 (0%) participants had pain classified as A, B, and C, respectively. The use of EC-760XP/L significantly reduced the degree of pain (*p* < 0.01).

**Table 2:** Outcomes of previous and current endoscopies.

	Ultrathin colonoscopy *n* = 39	Previous colonoscopy *n* = 39	*P-value
Cecal intubation time, min (IQR)	9.5 (5.3–12.0)	19 (15.8–25.0)	< 0.01
Cecal intubation rate, n	39 (100%)	30 (76.9%)	< 0.01
Observational time, n	10.0 (8.0–14.8)	12.0 (9.0–19.0)	0.42
Adenoma Detection, n (%)	23 (59.0%)	17 (43.6%)	0.18
Polyp Detection, n (%)	26 (66.7%)	20 (51.3%)	0.24
Boston Bowel preparation Scale, n (IQR)	9 (6.8–9)	9 (8–9)	0.68
Use of sedatives, n (%)	13 (33.3%)	10 (25.6%)	0.62
Antispasmodic, n (%)	33 (84.6%)	27 (69.2%)	0.17
Adverse events, n (%)	0	0	-
Pain scale (A / B / C), n (%)	36 (92.3%) /3 (7.7%) / 0 (0%)	16(41.0%)/19 (48.7%)/4 (10.3%)	< 0.01

^*^P-value was calculated using the χ^2^ test or Fisher’s exact test for categorical data.

P-value was calculated using a t test or the Mann–Whitney U test for continuous data.

IQR, interquartile range;

## Discussion

This study is a single-center, retrospective analysis that evaluates the use of the EC-760XP/L in patients who experienced difficulties with colonoscope insertion. Results showed that using this endoscope improved the cecal intubation rate, reduced cecal intubation time, and significantly reduced pain. To our knowledge, this is the first report that has demonstrated the usefulness of EC-760XP/L for patients with difficult colonoscope insertion. There have been several previous reports regarding colonoscopy using PCF-PQ260L (PQL), which is another small-diameter endoscope. Sato et al. used PQL as a rescue device in patients where the colonoscope could not be inserted during regular examination, and results showed that cecal intubation was achieved in 97.7% (42/43) of cases. They also showed that the mean pain score during the colonoscopy was significantly lower after the second PQL colonoscopy than during the first regular colonoscopy^18^. According to a report by Inoki et al.^5^, there were no significant differences in the cecal intubation rate, cecal intubation time, and ADR between the PQL and standard colonoscope use groups. However, a comparison between PQL examinations and standard colonoscopies in the same patient showed that PQL use resulted in a reduced mean cecal intubation time (7 min vs. 10 min, *p* < 0.01), as well as a significantly higher number of patients with less pain (66% vs. 20%, *p* < 0.01) and less use of sedation (48% vs. 25%, *p* < 0.01). Hamada et al. conducted a study in which female patients undergoing colonoscopy without sedatives were randomly assigned to either a group using PQL or a standard colonoscopy group. Their analysis revealed that the group using PQL experienced significantly less pain and demonstrated a higher willingness to undergo the next examination without sedatives compared to the standard colonoscopy group^19^. These results also indicate the usefulness of small-diameter long scopes in cases with insertion difficulties.

Similar to the present study, the report by Inoki et al.^5^ compared standard colonoscopy and PQL in the same patient; however, PQL was conducted by a specialist in a significantly higher number of cases, suggesting that the results may have been affected by differences in endoscopy techniques.

In our study, the inclusion criteria were specifically limited to cases where insertion was difficult during the previous examination, even when performed by an experienced endoscopist. Additionally, we focused on the same patients as those from the previous examination. We defined insertion difficulty as instances where the previous intubation time was ≥ 15 min, providing a more objective basis for analysis.

EC-760XP/L offers significant advantages, including LCI and a water jet function (sub-water supply function). A challenge in conventional narrow-band imaging and BLI is that water appears red, potentially impacting image clarity. In cases with insertion difficulties, inadequate pretreatment, insufficient suction of water, or residual feces are frequent, which may reduce the ADR of the enhanced image. Meanwhile, with LCI, the ADR can be expected to increase regardless of water influence, and the water jet function also enables the cleaning of the mucous membranes. Although our study did not demonstrate an additional effect on ADR or PDR compared to previous endoscopies, we observed encouraging results with an ADR of 59.0% and a PDR of 66.7%.

Furthermore, although there is a high risk of adverse events (such as adhesions) in cases with insertion difficulties, no adverse events, such as gastrointestinal perforation or abdominal pain, were confirmed in this study. Meanwhile, EC-760XP/L has a narrow forceps channel and long scope, so there is still room for improvement in terms of treatment. Future studies will need to consider ways to improve treatment.

The present study had several limitations. Firstly, it was a single-center, retrospective study, and as such, the difficulty experienced during colonoscopy is subjective. Additionally, due to learning bias, repeat colonoscopy tends to increase the success rate regardless of the type of scope used. Secondly, this study had a relatively small sample size of 39 patients, which may limit the statistical power and the ability to detect differences in secondary outcomes such as ADR and PDR. Thirdly, direct comparisons were not performed by conducting examinations with a standard scope following those with the EC-760XP/L. Another limitation of this study is the lack of blinding and randomization, which could introduce observer bias. This is particularly relevant when assessing subjective outcomes such as pain. Furthermore, each examination was conducted by different operators, which might have affected the consistency of the results.

Fourth, the role of sedatives warrants consideration. Although there were no significant differences in sedative use, their administration could potentially enhance the cecal intubation rate, reduce intubation times, and reduce pain. The degree of pain under sedation is unclear because we did not use scales such as the Visual Analogue Scale. In the future, conducting a multicenter, prospective, comparative study of EC-760XP/L in cases with insertion difficulties and accumulating cases is necessary.

## Data Availability

The datasets used and/or analyzed during the current study are available from the corresponding author on reasonable request.
